# Mechanistic Dissymmetry between Crystal Growth and Dissolution Drives Ratcheted Chiral Amplification

**DOI:** 10.1021/jacs.5c12199

**Published:** 2025-10-08

**Authors:** Sjoerd W. van Dongen, Jin Maeda, Bernard Kaptein, Pascal Cardinael, Adrian Flood, Gerard Coquerel, Willem L. Noorduin

**Affiliations:** † 55952 AMOLF, Science Park 104, 1098 XG, Amsterdam, The Netherlands; ‡ Univ Rouen Normandie, Normandie Univ, SMS UR 3233, F-76000 Rouen, France; § School of Energy Science and Engineering, 423058 Vidyasirimedhi Institute of Science and Technology, Rayong 21210, Thailand; ∥ 497749 InnoSyn, Urmonderbaan 22, 6167 RD Geleen, The Netherlands; ⊥ Van’t Hoff Institute for Molecular Sciences, University of Amsterdam, Science Park 904, 1098 XH Amsterdam, The Netherlands

## Abstract

Complete chiral amplification of the solid phase arises when mixtures of self-sorting enantiopure crystals undergo cycles of crystal growth and dissolution under solution-phase racemizing conditions. However, despite extensive studies and widespread use, the mechanism underlying such crystallization-induced deracemization remains insufficiently understood, hindering its optimization and broader application. Here, we experimentally dissect the individual contributions of crystal growth and dissolution and use a mass-balance to expose crystal dynamics. Regardless of the racemization rate, we always find a dissymmetry between the growth and the dissolution of the enantiomer populations. These experiments suggest that a fundamental difference between the mechanisms of crystal growth and dissolution enables a ratchet effect that drives chiral amplification. These insights advance our understanding of chiral crystallization mechanisms and provide guidance for optimizing crystallization-induced deracemizations, particularly by separately optimizing growth and dissolution steps to maximize the chiral amplification and deracemization efficiency.

## Graphical abstract

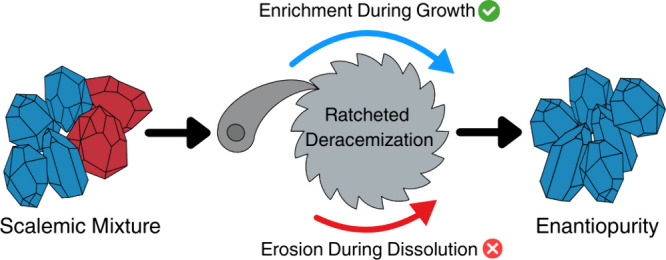

## Introduction

Chirality is a hallmark of life and a key challenge in chemistry.^1,2^ Isolating molecules of a desired chiral configuration is crucial for applications in pharmaceuticals, agrochemicals, and materials.^3–7^ The crystallization of racemic conglomerates,^8,9^ where chiral molecules self-sort into enantiopure crystals, offers an intrinsically stereoselective strategy to separate or deracemize mixtures of enantiomers into a desired configuration.^10–13^

Deracemization of the solid phase occurs when enantiopure crystals undergo cycles of crystal growth and dissolution while racemizing in solution (Figure 1a).^14–19^ The cycles of crystal growth and dissolution that drive deracemization may be implemented through temperature or solvent cycles or continuous attrition (Figure 1b).^20–31^ An initial enantioenrichment directs the deracemization process to the enantiomer of choice.

**Figure 1:**
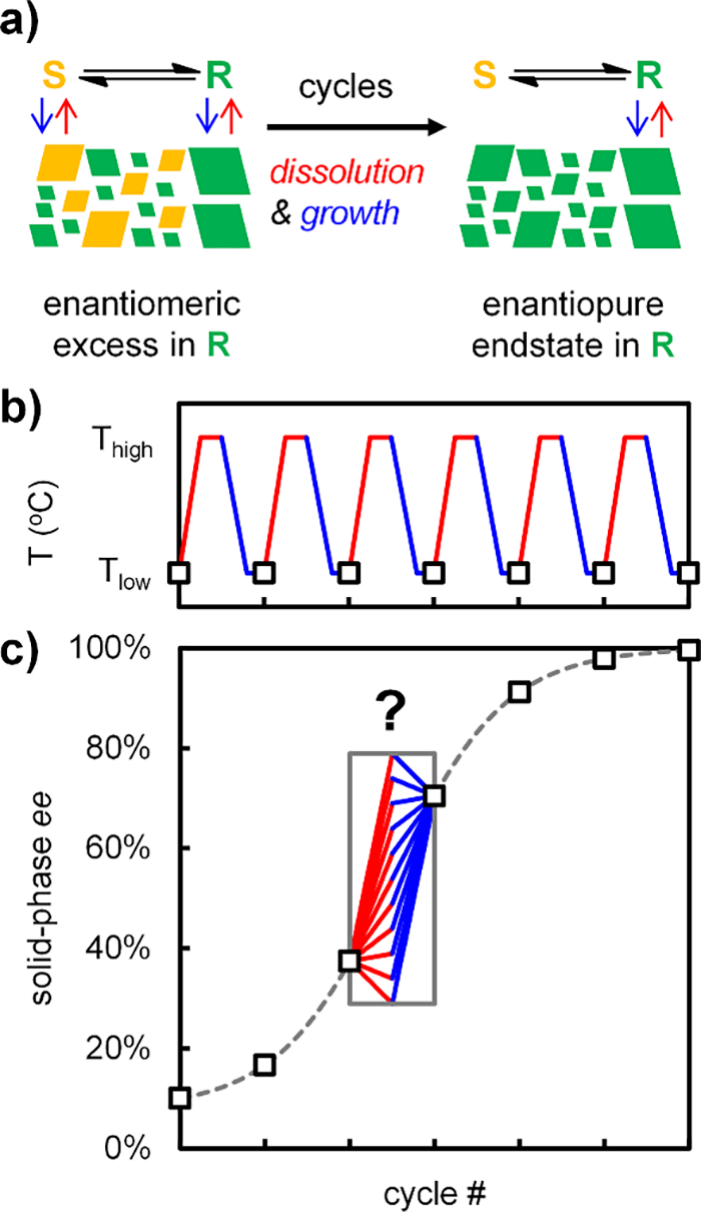
Crystallization-induced deracemization. (a) Crystal dissolution (red) and growth (blue) are coupled to racemization in solution to completely deracemize the solid phase of a slurry with an initial solid enantiomeric excess (*ee*). (b) Cyclic growth and dissolution through temperature cycles. (c) Every cycle increases the solid-phase *ee* (squares), but the individual contributions of dissolution and growth to chiral amplification are unknown.

Although crystallization-induced deracemization has already been demonstrated for an array of bioactive chiral molecules,^23,28,32–36^ the underlying mechanisms still pose questions of both fundamental and practical importance. Known is that each complete cycle of crystal growth and dissolution enantiomerically enriches the solid phase, and many theoretical models have been proposed.^37–50^ Unknown, however, is what occurs during the individual segments of crystal growth or dissolution, as this question has not yet been studied experimentally, beyond a demonstration of chiral amplification during growth.^48^ During crystal growth, the majority enantiomer has a higher growth rate, and racemization converts the minority into the majority to equalize supersaturation. It was recently hypothesized that the interplay between racemization and crystallization rate drives chiral amplification, as the inherently faster rate of dissolution compared to growth prevents racemization to fully neutralize the enantioenrichment obtained during growth.^47^ Population balance modeling for an idealized system has shown deracemization through this mechanism.^50^ Yet unclear, however, is how this mechanism can explain the deracemization for systems where racemization is very fast or instantaneous. Moreover, so far, no results have been reported to experimentally show the individual effects of crystal growth and dissolution for unequal and competing populations of crystals.

This work aims to experimentally and systematically dissect the contributions of dissolution and growth and identify how these separately enrich, erode, or preserve the solid-phase enantiomeric excess (Figure 1c). Using previously developed racemizable conglomerates paclobutrazol precursor **1** and *tert*-leucine precursor **2** (Scheme 1),^23,48,51–54^ we experimentally reveal the fundamental dissymmetry between asymmetric crystal growth and dissolution. This dissymmetry even exists when racemization kinetics are non-limiting and therefore is not caused by an interplay of crystallization and racemization rate alone, but likely results from a fundamental irreversibility between the mechanisms of crystal growth and dissolution. Through a full cycle of asymmetric dissolution and growth, this dissymmetry enables a ratchet-like effect that ultimately drives chiral amplification. This study challenges common tenets of crystallization-induced deracemization, shows that dissolution is antagonistic to chiral amplification, and exploits these insights to optimize deracemization processes.

**Scheme 1:**
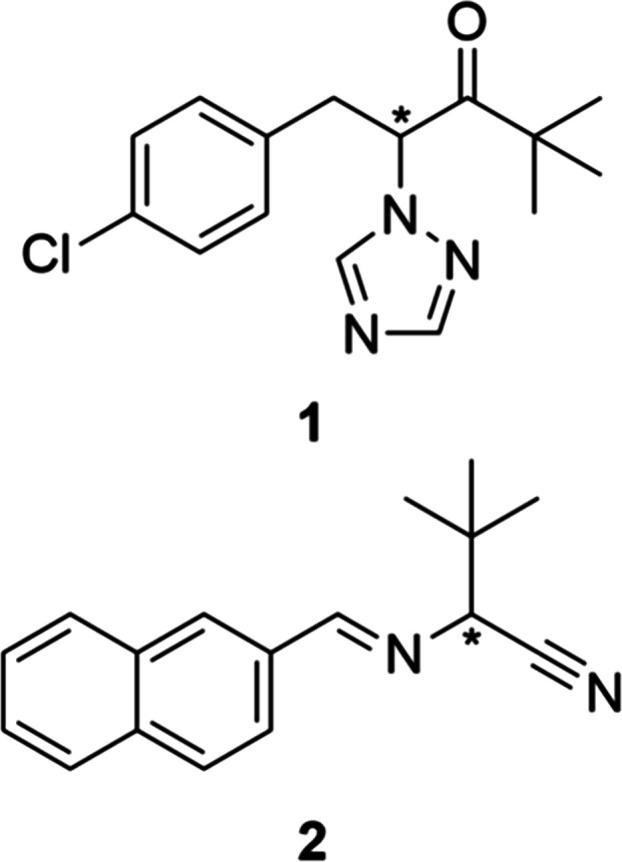
Racemizable Conglomerates **1** and **2** (* indicates chiral center)

## Results

To experimentally deconvolute cyclic dissolution and growth, we determined the evolution of the solid-phase enantiomeric excess (*ee*) after individual dissolution (heating) and growth (cooling) segments of a temperature cycle (Figure 2). Low initial solid loading and high solubility differences between the low and high temperatures of the cycle were used to increase the sensitivity to subtle effects in asymmetric crystallization.^48^ We prepared a slurry of conglomerate **1** at 40 °C in MeOH:water (60:40) with an initial ∼25% *ee* in (*R*)-**1** in the solid phase and started liquid-phase racemization by adding 0.1% w/v NaOH.^23,51,52^ We cycled between 40 and 55 °C and analyzed the solid-phase *ee* via chiral HPLC after heating-induced dissolution (1.5 °C/min) and after subsequent cooling-induced growth (0.5 °C/min), both after 10 min isothermal hold to fully reach equilibrium. Experiments were repeated for slurries with initial 50% and 90% *ee* in (*R*)-**1** in the solid phase.

**Figure 2:**
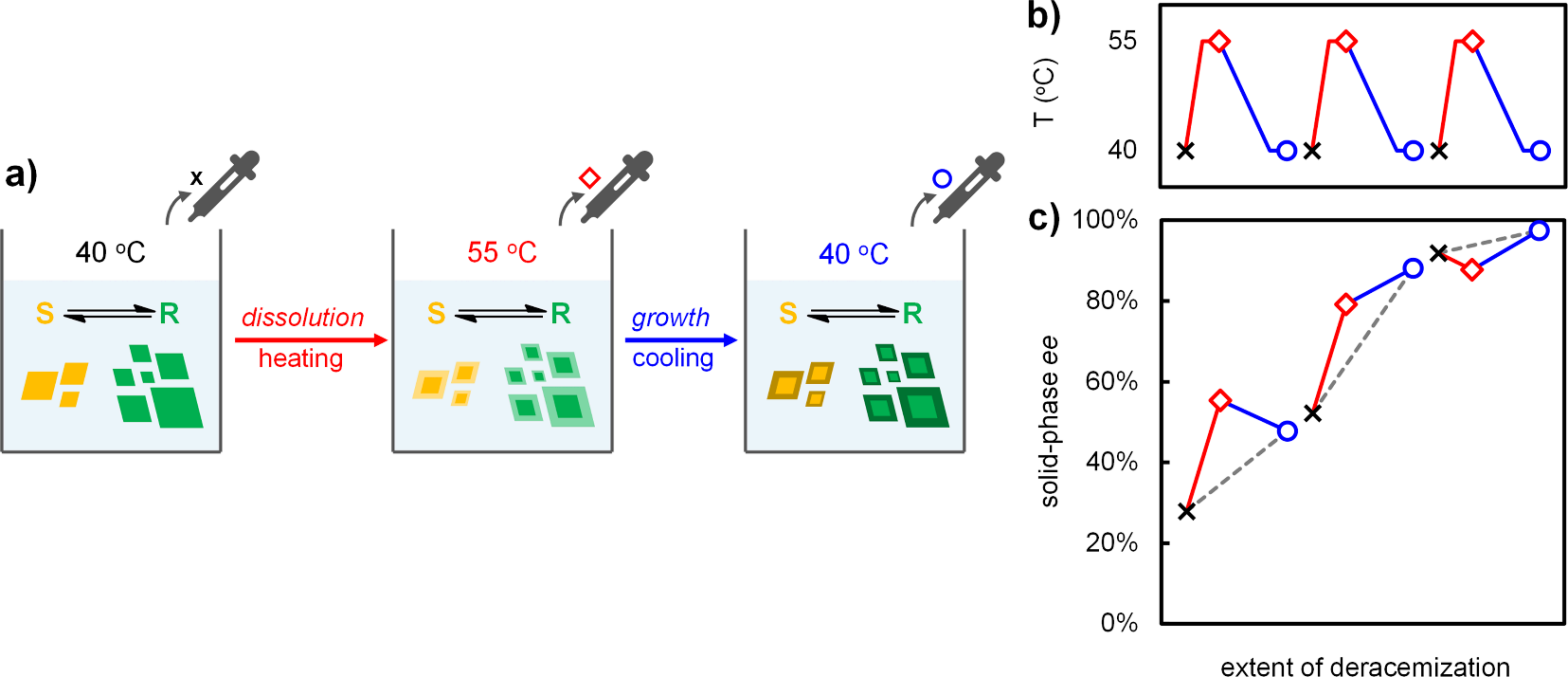
Deconvoluted temperature cycles for slurries with low, medium, and high initial *ee* values in (*R*)-**1**. (a) Solid-phase *ee* is determined by sampling before dissolution (black cross), after dissolution (red diamond), and after subsequent growth (blue circle). (b) Temperature profile with sampling moments indicated. (c) Solid-phase *ee* increases for every cycle (gray dashed line), although individual contributions of dissolution and growth vary (solid lines). Lines are a guide to the eye.

Figure 2c shows the solid-phase *ee* values after dissolution and growth, starting with different degrees of initial solid-phase enantiomeric enrichment. As expected, the solid-phase *ee* increases over each full cycle, confirming the net chiral amplification of the system. However, the extent of chiral amplification varies; cycles with low (∼25%) and high (∼90%) initial *ee* values show lower enrichment than cycles with medium (∼50%) initial *ee*, which is consistent with the sigmoidal characteristic of crystallization-induced deracemization kinetics.

The variation within the cycles is profound. For low and medium initial *ee*, Figure 2c shows an increase in solid-phase *ee* after dissolution, as expected. For high initial *ee*, however, dissolution is shown to decrease solid-phase *ee*, which is in contrast to the common view that dissolution always enriches solid-phase *ee.* This surprising decrease in solid-phase *ee* during dissolution is not affected by the heating rate or the duration of the isothermal hold and enhanced if the relative amount of dissolved solid is increased (see Supporting Information). Growth also allows for both enrichment and erosion: For low initial *ee*, the solid-phase *ee* decreases during growth, whereas for medium and high initial *ee* the solid-phase *ee* increases during growth. This experimental evidence for erosion during dissolution and enrichment during growth confirms that the major enantiomer may dissolve faster and grow faster than the minor enantiomer.^47,50^

Motivated by these results, we investigated how asymmetric crystallization causes the major enantiomer to dissolve and grow faster than the minor enantiomer. We therefore disentangled the deracemization process into completely isolated dissolution and growth steps (Figure 3a). Since crystals are only partly altered during dissolution and growth, we constructed a full mass balance, detailing exactly how much of each enantiomer is grown or dissolved. For this, we used conglomerate **2** (Scheme 1), which racemizes in the presence of 1,8-diazabicyclo[5.4.0]­undec-7-ene (DBU) and exhibits clean racemization kinetics without side-reactions.^48,53,54^ Since net chiral amplification is strongest at medium initial *ee* and boundary effects are minimized, we prepared initial solids with 50% *ee* in (*R*)-**2**. The initial solids were preground to ensure a uniform initial crystal size distribution and morphology.

**Figure 3:**
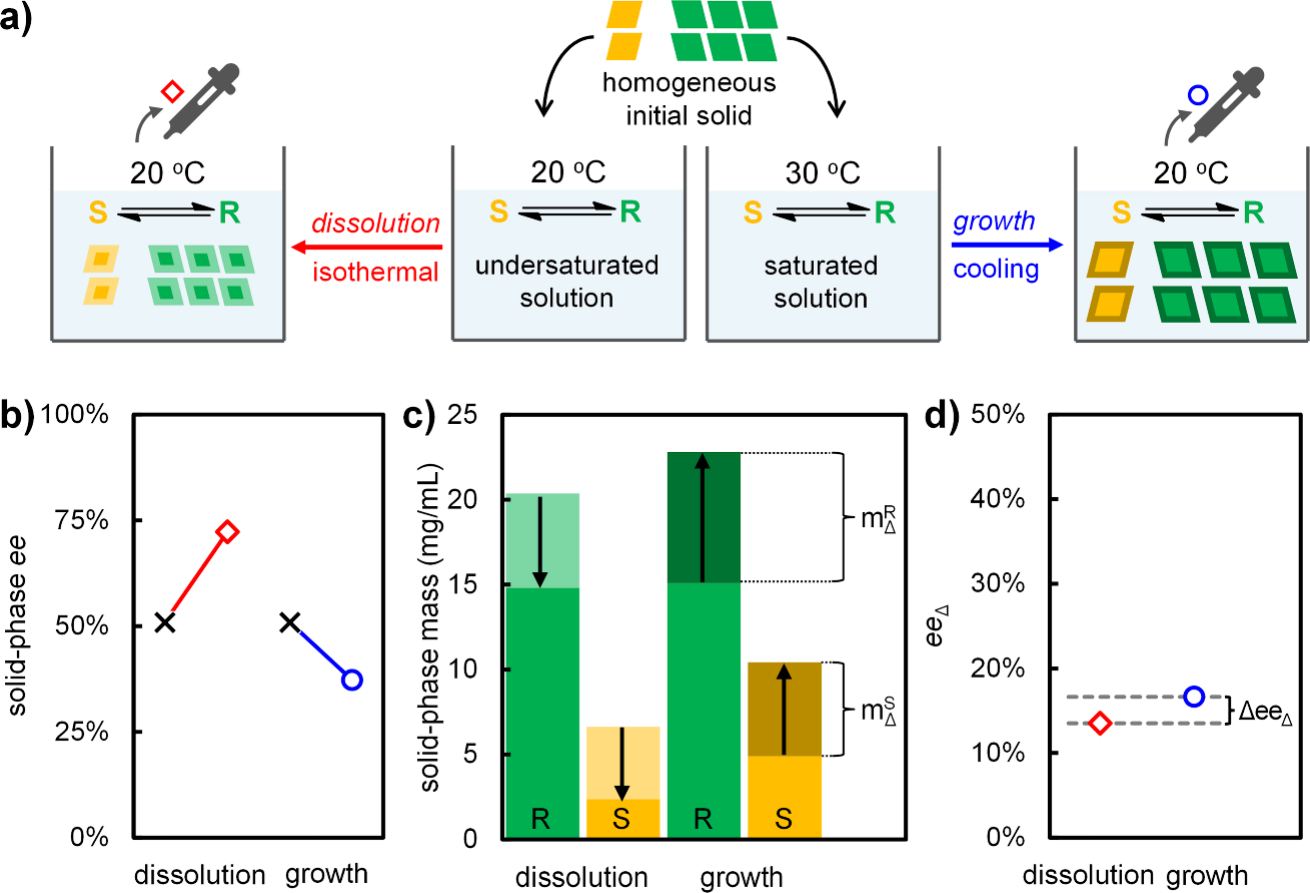
Dissymmetry between dissolution (red) and growth (blue) drives ratcheted chiral amplification. (a) Solid and liquid are analyzed after separate dissolution and growth starting from the same preground initial solid of 50% *ee* in (*R*)-**2**. (b) Absolute solid-phase *ee* increases after dissolution and decreases after growth. (c) Mass-balance constructed from liquid-phase concentrations shows the mass of grown and dissolved enantiomers (*m*_Δ_). (d) Positive *ee* of the dissolved and grown material (*ee*_Δ_) shows that the major enantiomer dissolves and grows faster than the minor enantiomer. Enrichment during growth outweighs erosion during dissolution (Δ*ee*_Δ_ > 0), a dissymmetry that enables ratcheted chiral amplification. Data are provided in the Supporting Information.

For dissolution, while at 20 °C, a racemizing undersaturated solution of (*R,S*)-**2** in MeOH (10 μL/mL DBU) was added to the preground initial solid. The resulting slurry was shaken in the presence of soft PTFE spheres to homogenize while minimizing attrition and secondary nucleation effects.^29^ After 90 min post-dissolution equilibration at 20 °C, the solid-phase *ee* was analyzed. Simultaneously, liquid-phase samples were taken to track enantiomer concentrations. For growth, while at 30 °C, a racemizing saturated solution of (*R,S*)-**2** in MeOH was added to a new aliquot of preground initial solid, which was slowly cooled back to 20 °C (0.11 °C/min) to avoid nucleation. Solid and liquid phases were sampled after 30 min post-growth equilibration at 20 °C, when crystallization had completed as confirmed via mass-balance. Since both dissolution and growth experiments ended at room temperature (20 °C), undesired crystallization during sampling was negligible.

Figure 3b shows the absolute solid-phase *ee* after independent dissolution and growth from identical preground initial solids. At first glance, the experiment seems to confirm the common view that solid-phase *ee* increases during dissolution and decreases during growth. However, these absolute changes in solid-phase *ee* can be deceiving. For instance, dissolving more majority than minority enantiomer can still cause an increase in solid-phase *ee*, but it effectively erodes the overall system *ee* through racemization. Exploiting our mass-balance, we therefore plot the initial mass and calculate the change in mass (*m*_Δ_) for both enantiomer populations using their initial and final liquid-phase concentrations (Figure 3c, *m*_Δ_ indicated using arrows).

Figure 3c shows that both enantiomers decrease in total solid mass during dissolution and increase in solid mass during growth but does not show how the initial imbalance between the enantiomer populations translates to asymmetric crystallization kinetics. To reveal this, we determined the *ee* of the portion of the crystals that is cumulatively removed from the crystals during dissolution and is cumulatively added to the crystals during growth (Figure 3d, *ee*_Δ_ = (*m*_Δ_^R^ – *m*_Δ_^S^)/(*m*_Δ_^R^ + *m*_Δ_^S^)).

To interpret *ee*_Δ_, we realize that when *ee*_Δ_ = 0, growth and dissolution are racemic (i.e., equal amounts of *R* and *S* are grown onto or dissolved from the initial solid, as under nonracemizing conditions) and no net amplification occurs over a full cycle. When *ee*_Δ_ = *ee*_0_ = 50%, growth or dissolution rates are proportional to the initial mass ratio of the enantiomers. Since the determined values of *ee*_Δ_ are positive for both dissolution and growth (Figure 3d), the major enantiomer consistently dissolves and grows faster than the minor enantiomer. Despite the use of identical initial solids, however, Figure 3d also shows that the value of *ee*_Δ_ differs for dissolution and growth and shows that the asymmetry is actually stronger during growth: *ee*_Δ_ = 17% for growth, while *ee*_Δ_ = 14% for dissolution. This difference experimentally shows that liquid-phase racemization during growth enhances chiral amplification, while racemization during dissolution is counterproductive, as the major enantiomer molecules are effectively racemized. Nevertheless, over a full cycle growth-driven amplification outweighs dissolution-induced erosion. These results thus experimentally reveal how chiral dissolution and growth are dissymmetric processes, enabling a ratchet effect that drives net chiral amplification over a full cycle.

The origin of this dissymmetry could be the coupled kinetics of racemization and crystallization, when crystals dissolve faster than they grow,^47,50^ but may also stem directly from fundamental differences between the mechanisms of crystal growth and dissolution. We therefore systematically varied the racemization rate to investigate how the observed effects depend on the interplay between rates of dissolution, growth, and racemization. Since the racemization rate of **2** is linearly dependent on the catalyst concentration ([DBU]),^55^ we repeated the previous experiment at various concentrations of DBU and plot the results (Figure 4).

**Figure 4:**
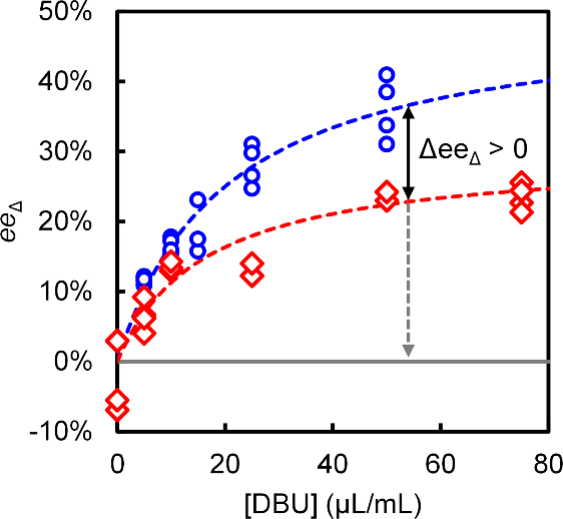
Dissolution (red) and growth (blue) are dissymmetric at every racemization rate (∝ [DBU]). Chiral amplification efficiency (∝ Δ*ee*_Δ_, solid black arrow) increases with the racemization rate. Chiral amplification efficiency can be maximized by switching off racemization during dissolution, such that *ee*_Δ_ = 0 (dashed gray arrow). Lines are a guide to the eye.

Without racemization, we observe the expected racemic growth and dissolution (*ee*_Δ_ = 0). With racemization, we observe a continuously increasing *ee*_Δ_ for both growth and dissolution, with the *ee*_Δ_ for growth always above that for dissolution. The larger the difference between the *ee*_Δ_ for dissolution and growth (Δ*ee*_Δ_), the stronger the ratchet effect, resulting in a higher chiral amplification efficiency. As expected from previous reports,^27,38,40,55,56^ increasing the racemization rate will thus generally increase deracemization kinetics by increasing Δ*ee*_Δ_.

Figure 4 shows that increasing the racemization rate increases *ee*_Δ_, until the racemization rate approaches a plateau. This effect has previously been reported for chiral crystal growth^48^ and thus also holds for dissolution. In this plateau, crystal growth and dissolution kinetics are much slower than the racemization kinetics. Hence, akin to Michaelis–Menten kinetics in biochemistry, racemization has become non-limiting. The fact that Figure 4 shows that *ee*_Δ_ for growth is systematically higher than *ee*_Δ_ for dissolution implies that amplification of the major enantiomer during growth always outweighs its erosion during dissolution: Net chiral amplification always occurs. This independence of racemization rate implies that the observed dissymmetry between chiral growth and dissolution is not merely caused by the kinetic interplay of racemization and crystallization but, at its core, rooted in a fundamental difference between the mechanisms of crystal growth and dissolution. This would explain why the dissymmetry that drives deracemization persists, even when racemization kinetics are no longer limiting.

The plateau in Figure 4 has practical consequences: there is a limit to the beneficial effects that can be gained by increasing the racemization kinetics. We realize, however, that increasing the racemization rate is not the only way to increase the efficiency of chiral amplification. Counterintuitively, chiral amplification efficiency can be increased further by switching racemization off entirely during dissolution. Without racemization, *ee*_Δ_ becomes 0 during dissolution, while the original *ee*_Δ_ during growth is retained, thereby maximizing Δ*ee*_Δ_. To explore this idea, we extended a basic model previously introduced for asymmetric crystal growth,^48^ by including a contribution for asymmetric dissolution. In short, we used the values of *ee*_Δ_ in Figure 4 to express asymmetric crystallization through empirical amplification factors for growth and dissolution (see Supporting Information for details). Although this qualitative model ignores many complexities of the deracemization process, it indeed predicts that switching off racemization during dissolution markedly increases the deracemization efficiency (Figure 5a). Moreover, the model visualizes that switching off racemization during growth would lead to a complete loss of the initial solid-phase enantiomeric excess. These predictions thus suggest that individually optimizing growth and dissolution can maximize chiral amplification efficiency.

**Figure 5:**
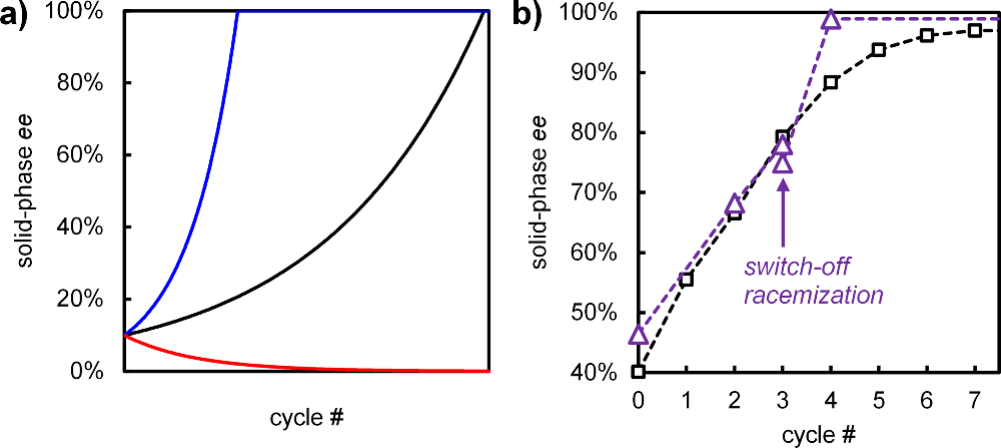
Increased deracemization efficiency through on/off switching of racemization. (a) Compared to a regular cycle (black), racemizing only during growth accelerates deracemization (blue), while racemizing only during dissolution causes racemization of the solid phase (red). (b) One-time switching off of racemization before the final dissolution step (purple triangles; racemization is on during final growth step) in the temperature cycling of **1** increases deracemization efficiency compared to regular cycles (black squares).

Repetitively switching on and off racemization is thus highly desirable but often hardly possible or practical. However, switching off racemization once, e.g., by quenching the catalyst, is generally feasible. Since deracemization kinetics slow significantly near the end, it may be beneficial to switch off racemization before a final dissolution step. To experimentally demonstrate this idea, we used conglomerate **1**, because racemization catalyst NaOH can be quenched by the addition of HCl. Starting with a solid phase of 40–45% *ee* in (*R*)-**1** and 0.1% w/v NaOH, we performed three cycles to reach 75% *ee* in the solid. We then switched off racemization by adding 1.1 equiv of 6 M HCl before performing a final dissolution step. After re-adding the racemization catalyst (0.2% w/v NaOH) a virtually enantiopure solid phase was obtained after growth (99% *ee* in (*R*)-**1**, Figure 5b). In contrast, the experiment wherein we kept racemization switched on required four more cycles to reach a similar solid-phase *ee* (97%). Hence, even switching off racemization only once already yields a higher enantiopurity within fewer cycles and decreases the time to deracemization by about 25% (Figure 5b).

## Discussion, Summary, and Outlook

Our findings bring surprising new insights to the fundamental mechanistic understanding of crystallization-induced deracemizations.

First, our results impact the debate on the mechanistic role of crystal size. Crystal-size effects and size-dependent solubility have been often proposed as central driving forces for chiral amplification,^39,41,44,49,57^ although these mechanisms have received criticism from other theorists.^47,58^ Here, we minimized the effect of crystal-size effects by using constant and homogeneous seeds. Nonetheless, we do observe the dissymmetric growth and dissolution that drive chiral amplification. Our results suggest that chiral amplification is not a result of crystal size effects alone, although such effects can surely modulate the degree of amplification.

Second, for the first time, we experimentally reveal the individually different contributions of crystal growth and dissolution and show that their dissymmetry causes a ratcheting effect that drives chiral amplification. Paradoxically, although dissolution may increase and growth may decrease the absolute solid-phase *ee*, a full mass balance shows that growth amplifies and dissolution erodes the enantiomeric enrichment of the entire system. These experimental results emphasize the proposed role of crystallization dynamics^47,48,50^ and contrast with previous views rooted in equilibrium thermodynamics.^45,59^ As such, our study demonstrates how experimentally deconvoluting the effects of crystal growth and dissolution for populations of crystals under racemizing conditions may not only help to validate assumptions on the mechanism,^37,39,41–47,49,50^ but also bring new mechanistic insights in asymmetric crystallization.

Third, we zoom in on the source of the dissymmetry between growth and dissolution that enables ratcheted chiral amplification. Although the interplay between rates of racemization and crystallization indeed modulates the degree of chiral amplification when racemization is relatively slower during dissolution than during growth,^47,50^ we experimentally find that chiral amplification also occurs when racemization is very fast and non-limiting. This finding suggests that it is the fundamental difference between the mechanisms of growth and dissolution, not just their interplay with the rate of racemization, that is the core driver of crystallization-induced chiral amplification. Such a mechanistic dissymmetry could also explain deracemization in achiral systems (e.g., NaClO_3_ and (H_2_NCH_2_CH_2_NH_2_)·H_2_SO_4_), where racemization is instantaneous.^17,18^

The mechanistic differences between crystal growth and dissolution are manifold. Beyond modes of attachment and detachment of molecules at crystal surfaces,^60–63^ important factors to consider are stereoselective incorporation of clusters and oriented attachment,^37,40,64–66^ ripening and agglomeration mechanisms,^39,67,68^ and a form of stereoselective secondary nucleation.^69–71^ Also population-level effects such as stochasticity (e.g., chiral flipping and growth rate dispersion) and non-ideal solution behavior of enantiomers may cause asymmetric crystallization.^44,70,72^ The predominant dissymmetry may depend on the crystallization conditions (e.g., supersaturation and attrition) and crystallization characteristics of the species (e.g., morphology, surface tension, and binding strength), and several of these mechanisms may be at play simultaneously. In this study, primary and secondary nucleation were aimed to be minimal, and crystallization proceeded at low supersaturation. Growth and dissolution of crystals always occur, even without attrition or explicit fluctuations in temperature or concentration,^42^ and the effects demonstrated here will be prevalent for all chiral crystals under racemizing conditions. Understanding growth and dissolution processes on the surface of a single crystal and translating those across interacting populations of many crystals will be key in unraveling the whole mechanism of chiral amplification through asymmetric crystallization.

Our results hold important practical lessons for designing and performing crystallization-induced deracemizations: (1) optimize dissolution and growth separately for maximum efficiency; (2) minimize racemization during dissolution and dissolve as fast as possible; and (3) maximize racemization during growth. The implementation of the cycles ideally should optimize the amount of cycled material per unit time.^73^ An effective approach would be to push the system away from equilibrium, thereby achieving simultaneous and continuous growth/dissolution cycles, as in the case of grinding and spatial temperature cycling.^22,74,75^ Moreover, we confirm a potential trap:^50^ Racemization reactions that proceed at different rates during growth and dissolution, e.g. due to strong temperature dependence of its reaction kinetics, can hinder chiral amplification and may even lead to solid-phase racemization rather than deracemization (i.e., when Δ*ee*_Δ_ < 0).

These insights can also aid in comparing and choosing different deracemization strategies. Solvent cycling,^29,30^ for instance, follows many of these practical lessons: The dissolution rate is maximized through instant re-addition of solvent; racemization is maximized during growth through slow evaporation; both growth and dissolution occur at equal temperatures and thus experience equal racemization rates.

To maximize chiral amplification, however, it will be required to sequentially switch on and off racemization. We therefore foresee the development of mechanical or chemical on/off switches, racemization based on electrochemistry and photochemistry, and exploiting gradients in experimental reactors. Also, flow chemistry or immobilized (bio)­catalysts could be utilized in a separate racemization loop that is activated or deactivated on demand.^28,76,77^ A ratchet effect may then also be exploited to deracemize thermodynamically stable racemic compounds.^13,78–80^

## Supplementary Material



## Notes

Experimental details (materials, methods, solubility data, supplementary data on deconvoluted temperature cycles, mass balance and *ee*_Δ_ determination), thermodynamic analysis using phase diagrams, model for asymmetric growth and dissolution (PDF)

## References

[1] Noyori R. (2002). Asymmetric
catalysis: science and opportunities (Nobel lecture). Angew. Chem., Int. Ed..

[2] Comprehensive Chirality, 1st ed.; Carreira, E. M. , Yamamoto, H. , Eds.; Elsevier: Amsterdam, 2012.

[3] List B., Yang J. W. (2006). The organic approach to asymmetric
catalysis. Science.

[4] Palmans A. (2017). Deracemisations
under kinetic and thermodynamic control. Mol.
Syst. Des. Eng..

[5] Banerjee-Ghosh K., Ben Dor O., Tassinari F., Capua E., Yochelis S., Capua A., Yang S.-H., Parkin S. S., Sarkar S., Kronik L. (2018). Separation of enantiomers by their enantiospecific
interaction with achiral magnetic substrates. Science.

[6] Crassous J., Fuchter M. J., Freedman D. E., Kotov N. A., Moon J., Beard M. C., Feldmann S. (2023). Materials for chiral light control. Nat. Rev. Mater..

[7] McVicker R. U., O’Boyle N. M. (2024). Chirality
of new drug approvals (2013–2022):
trends and perspectives. J. Med. Chem..

[8] Jacques, J. ; Collet, A. ; Wilen, S. H. ; Collet, A. Enantiomers, Racemates, and Resolutions; Wiley: New York, 1981.

[9] Walsh M. P., Barclay J. A., Begg C. S., Xuan J., Johnson N. T., Cole J. C., Kitching M. O. (2022). Identifying
a hidden conglomerate
chiral pool in the CSD. JACS Au.

[10] Brands K. M., Davies A. J. (2006). Crystallization-induced diastereomer transformations. Chem. Rev..

[11] Lorenz H., Seidel-Morgenstern A. (2014). Processes
to separate enantiomers. Angew. Chem., Int.
Ed..

[12] Buhse T., Cruz J.-M., Noble-Teran M. E., Hochberg D., Ribo J. M., Crusats J., Micheau J.-C. (2021). Spontaneous deracemizations. Chem. Rev..

[13] Pinetre C., van Dongen S. W., Brandel C., Léonard A.-S., Charpentier M. D., Dupray V., Oosterling K., Kaptein B., Leeman M., Kellogg R. M. (2025). Enantiopurity
by directed evolution of crystal stabilities and nonequilibrium crystallization. J. Am. Chem. Soc..

[14] Havinga E. (1954). Spontaneous
formation of optically active substances. Biochim.
Biophys. Acta.

[15] Kondepudi D. K., Kaufman R. J., Singh N. (1990). Chiral symmetry breaking in sodium
chlorate crystallization. Science.

[16] McBride J. M., Carter R. L. (1991). Spontaneous resolution by stirred crystallization. Angew. Chem., Int. Ed..

[17] Viedma C. (2005). Chiral Symmetry
Breaking During Crystallization: Complete Chiral Purity Induced by
Nonlinear Autocatalysis and Recycling. Phys.
Rev. Lett..

[18] Cheung P. S. M., Gagnon J., Surprenant J., Tao Y., Xu H., Cuccia L. A. (2008). Complete asymmetric amplification
of ethylenediammonium
sulfate using an abrasion/grinding technique. Chem. Commun..

[19] Noorduin W. L., Izumi T., Millemaggi A., Leeman M., Meekes H., Van Enckevort W. J., Kellogg R. M., Kaptein B., Vlieg E., Blackmond D. G. (2008). Emergence of a single solid chiral state from a nearly
racemic amino acid derivative. J. Am. Chem.
Soc..

[20] Leeman M., Noorduin W. L., Millemaggi A., Vlieg E., Meekes H., van Enckevort W. J., Kaptein B., Kellogg R. M. (2010). Efficient Havinga–Kondepudi
resolution of conglomerate amino acid derivatives by slow cooling
and abrasive grinding. CrystEngComm.

[21] Noorduin W., Van Der Asdonk P., Bode A., Meekes H., Van Enckevort W., Vlieg E., Kaptein B., Van Der Meijden M., Kellogg R., Deroover G. (2010). Scaling up attrition-enhanced deracemization
by use of an industrial bead mill in a route to Clopidogrel (Plavix). Org. Process Res. Dev..

[22] Viedma C., Cintas P. (2011). Homochirality beyond
grinding: deracemizing chiral
crystals by temperature gradient under boiling. Chem. Commun..

[23] Suwannasang K., Flood A., Rougeot C., Coquerel G. (2013). Using programmed heating–cooling
cycles with racemization in solution for complete symmetry breaking
of a conglomerate forming system. Cryst. Growth.
Des..

[24] Li W. W., Spix L., De Reus S. C., Meekes H., Kramer H. J., Vlieg E., Ter Horst J. H. (2016). Deracemization of a racemic compound
via its conglomerate-forming salt using temperature cycling. Cryst. Growth. Des..

[25] Breveglieri F., Maggioni G. M., Mazzotti M. (2018). Deracemization
of NMPA via temperature
cycles. Cryst. Growth. Des..

[26] Cameli F., Xiouras C., Stefanidis G. D. (2018). Intensified
deracemization via rapid
microwave-assisted temperature cycling. CrystEngComm.

[27] Intaraboonrod K., Lerdwiriyanupap T., Hoquante M., Coquerel G., Flood A. E. (2020). Temperature
cycle induced deracemization. Mendeleev Commun..

[28] Valenti G., Tinnemans P., Baglai I., Noorduin W. L., Kaptein B., Leeman M., Ter Horst J. H., Kellogg R. M. (2021). Combining incompatible
processes for deracemization of a Praziquantel derivative under flow
conditions. Angew. Chem..

[29] van
Dongen S. W., Baglai I., Leeman M., Kellogg R. M., Kaptein B., Noorduin W. L. (2023). Rapid deracemization through solvent
cycling: proof-of-concept using a racemizable conglomerate clopidogrel
precursor. Chem. Commun..

[30] Intaraboonrod K., Flood A. E. (2023). A Novel Strategy
for Deracemization Using Periodic
Fluctuations of Concentration. Chem. Eng. Technol..

[31] Gieling J., Wéry G., Lopes C., de Meester J., Brandel C., Cartigny Y., Leyssens T., Baier D. M. (2025). Mechanochemical
Deracemization: A Sustainable Approach to Enantiopurity. Chem.—Eur. J..

[32] van
der Meijden M. W., Leeman M., Gelens E., Noorduin W. L., Meekes H., van Enckevort W. J., Kaptein B., Vlieg E., Kellogg R. M. (2009). Attrition-enhanced deracemization in the synthesis
of clopidogrel-a practical application of a new discovery. Org. Process Res. Dev..

[33] Noorduin W. L., Kaptein B., Meekes H., van Enckevort W. J., Kellogg R. M., Vlieg E. (2009). Fast attrition-enhanced
deracemization
of naproxen by a gradual in situ feed. Angew.
Chem., Int. Ed..

[34] Baglai I., Leeman M., Kellogg R. M., Noorduin W. L. (2019). A Viedma ripening
route to an enantiopure building block for Levetiracetam and Brivaracetam. Org. Biomol. Chem..

[35] Shimizu W., Uemura N., Yoshida Y., Mino T., Kasashima Y., Sakamoto M. (2020). Attrition-enhanced
deracemization and absolute asymmetric
synthesis of flavanones from prochiral precursors. Cryst. Growth. Des..

[36] Oketani R., Naito R., Hisaki I. (2024). Semicontinuous
temperature cycle-induced
deracemization using an axially chiral naphthamide. Org. Process Res. Dev..

[37] Uwaha M. (2004). A model for
complete chiral crystallization. J. Phys. Soc.
Jpn..

[38] Noorduin W. L., Meekes H., van Enckevort W., Millemaggi A., Leeman M., Kaptein B., Kellogg R., Vlieg E. (2008). Complete Deracemization
by Attrition-Enhanced Ostwald Ripening Elucidated. Angew. Chem., Int. Ed..

[39] Noorduin W. L., Meekes H., Bode A. A., van Enckevort W. J., Kaptein B., Kellogg R. M., Vlieg E. (2008). Explanation
for the
emergence of a single chiral solid state during attrition-enhanced
Ostwald ripening: survival of the fittest. Cryst.
Growth. Des..

[40] Noorduin W. L., van Enckevort W. J., Meekes H., Kaptein B., Kellogg R. M., Tully J. C., McBride J. M., Vlieg E. (2010). The driving mechanism
behind attrition-enhanced deracemization. Angew.
Chem., Int. Ed..

[41] Iggland M., Mazzotti M. (2011). A population balance
model for chiral resolution via
Viedma ripening. Cryst. Growth. Des..

[42] Hein J. E., Huynh Cao B., Viedma C., Kellogg R. M., Blackmond D. G. (2012). Pasteur’s
tweezers revisited: on the mechanism of attrition-enhanced deracemization
and resolution of chiral conglomerate solids. J. Am. Chem. Soc..

[43] Suwannasang K., Coquerel G., Rougeot C., Flood A. E. (2014). Mathematical modeling
of chiral symmetry breaking due to differences in crystal growth kinetics. Chem. Eng. Technol..

[44] Uchin R., Suwannasang K., Flood A. E. (2017). Model of Temperature Cycle-Induced
Deracemization via Differences in Crystal Growth Rate Dispersion. Chem. Eng. Technol..

[45] Belletti, G. Solid State Deracemization. Viedma ripening versus temperature cycling; Radboud University: Nijmegen, 2021.

[46] Dutta S., Yun Y., Widom M., Gellman A. J. (2021). 2D Ising Model for Adsorption-induced
Enantiopurification of Racemates. ChemPhysChem.

[47] Uwaha M., Katsuno H. (2022). Mechanism of chirality conversion of crystals by Viedma
ripening and temperature cycling. J. Cryst.
Growth.

[48] van
Dongen S. W., Ahlal I., Leeman M., Kaptein B., Kellogg R. M., Baglai I., Noorduin W. L. (2023). Chiral amplification
through the interplay of racemizing conditions and asymmetric crystal
growth. J. Am. Chem. Soc..

[49] Zhang B., Coquerel G., Kim W.-S. (2023). Isothermal
deracemization of sodium
chlorate in an agitated reactor: the effect of crystal size variables. Cryst. Growth. Des..

[50] Deck L.-T., Hosseinalipour M. S., Mazzotti M. (2024). Exact and Ubiquitous Condition for
Solid-State Deracemization in Vitro and in Nature. J. Am. Chem. Soc..

[51] Black S., Williams L., Davey R., Moffatt F., Jones R., McEwan D., Sadler D. (1989). The preparation
of enantiomers of
paclobutrazol: a crystal chemistry approach. Tetrahedron.

[52] Maeda J., Cardinael P., Flood A., Coquerel G. (2024). Improved Experimental
Yield of Temperature-Cycle-Induced Deracemization (TCID) with Cooling
and Crystal Washing: Application of TCID for the Industrial Scale. Crystals.

[53] Baglai I., Leeman M., Wurst K., Kaptein B., Kellogg R. M., Noorduin W. L. (2018). The Strecker reaction coupled to Viedma ripening: a
simple route to highly hindered enantiomerically pure amino acids. Chem. Commun..

[54] Breveglieri F., Baglai I., Leeman M., Noorduin W. L., Kellogg R. M., Mazzotti M. (2020). Performance analysis
and model-free design of deracemization
via temperature cycles. Org. Process Res. Dev..

[55] Breveglieri F., Mazzotti M. (2019). Role of racemization kinetics in the deracemization
process via temperature cycles. Cryst. Growth.
Des..

[56] Oketani R., Hoquante M., Brandel C., Cardinael P., Coquerel G. (2018). Practical role of racemization rates in deracemization
kinetics and process productivities. Cryst.
Growth. Des..

[57] Bodák B., Maggioni G. M., Mazzotti M. (2018). Population-based mathematical model
of solid-state deracemization via temperature cycles. Cryst. Growth. Des..

[58] Ricci F., Stillinger F. H., Debenedetti P. G. (2013). A computational investigation of
attrition-enhanced chiral symmetry breaking in conglomerate crystals. J. Chem. Phys..

[59] Coquerel G. (2015). Solubility
of chiral species as function of the enantiomeric excess. J. Pharm. Pharmacol..

[60] Weissbuch I., Addadi L., Lahav M., Leiserowitz L. (1991). Molecular
recognition at crystal interfaces. Science.

[61] Horvath J. D., Koritnik A., Kamakoti P., Sholl D. S., Gellman A. J. (2004). Enantioselective
separation on a naturally chiral surface. J.
Am. Chem. Soc..

[62] Snyder R. C., Doherty M. F. (2007). Faceted crystal
shape evolution during dissolution
or growth. AIChE J..

[63] Snyder R. C., Studener S., Doherty M. F. (2007). Manipulation
of crystal shape by
cycles of growth and dissolution. AIChE J..

[64] Spix L., Engwerda A. H., Meekes H., van Enckevort W. J., Vlieg E. (2016). Persistent reverse enantiomeric excess
in solution during Viedma
ripening. Cryst. Growth. Des..

[65] Viedma C., McBride J. M., Kahr B., Cintas P. (2013). Enantiomer-specific
oriented attachment: formation of macroscopic homochiral crystal aggregates
from a racemic system. Angew. Chem., Int. Ed..

[66] Katsuno H., Uwaha M. (2016). Mechanism of chirality conversion by periodic change of temperature:
Role of chiral clusters. Phys. Rev. E.

[67] Zhang B., Park B. J., Coquerel G., Kim W.-S. (2025). Agglomeration of
Homochiral sodium chlorate crystals under near-equilibrium conditions. Powder Technol..

[68] Shtukenberg A. G., García-Ruiz J.
M., Kahr B. (2021). Punin Ripening
and
the Classification of Solution-Mediated Recrystallization Mechanisms. Cryst. Growth. Des..

[69] Saito Y., Hyuga H. (2008). Chiral crystal growth
under grinding. J. Phys.
Soc. Jpn..

[70] Skrdla P. J. (2011). Kinetics
and thermodynamics of efficient chiral symmetry breaking in nearly
racemic mixtures of conglomerate crystals. Cryst.
Growth. Des..

[71] Cameli F., Ter Horst J. H., Steendam R. R., Xiouras C., Stefanidis G. D. (2020). On the
effect of secondary nucleation on deracemization through temperature
cycles. Chem.—Eur. J..

[72] Choi H. S., Oh I. H., Zhang B., Coquerel G., Kim W.-S., Park B. J. (2024). Chiral Flipping
in Viedma Deracemization. J. Phys. Chem. Lett..

[73] Wu Z., Yang S., Wu W. (2016). Application of temperature cycling
for crystal quality control during crystallization. CrystEngComm.

[74] Viedma C. (2007). Chiral symmetry
breaking and complete chiral purity by thermodynamic-kinetic feedback
near equilibrium: implications for the origin of biochirality. Astrobiology.

[75] Suwannasang K., Flood A. E., Coquerel G. (2016). A novel design approach to scale
up the temperature cycle enhanced deracemization process: coupled
mixed-suspension vessels. Cryst. Growth. Des..

[76] Intaraboonrod K., Harriehausen I., Carneiro T., Seidel-Morgenstern A., Lorenz H., Flood A. E. (2021). Temperature cycling induced deracemization
of dl-asparagine monohydrate with immobilized amino acid racemase. Cryst. Growth. Des..

[77] Kwan M. H., Breen J., Bowden M., Conway L., Crossley B., Jones M. F., Munday R., Pokar N. P., Screen T., Blacker A. J. (2021). Continuous flow
chiral amine racemization applied to
continuously recirculating dynamic diastereomeric crystallizations. J. Org. Chem..

[78] Viedma C., Ortiz J. E. (2021). A new twist in eutectic
composition: deracemization
of a racemic compound amino acid by Viedma ripening and temperature
fluctuation. Isr. J. Chem..

[79] Borsley S., Gallagher J. M., Leigh D. A., Roberts B. M. (2024). Ratcheting synthesis. Nat. Rev. Chem..

[80] Borsley S., Leigh D. A., Roberts B. M. (2024). Molecular
ratchets and kinetic asymmetry:
giving chemistry direction. Angew. Chem., Int.
Ed..

