# Supplementary material for: Mechanistic Dissymmetry between Crystal Growth and Dissolution Drives Ratcheted Chiral Amplification
Source: J Am Chem Soc. 2025 Oct 8;147(42):38508–15. doi: 10.1021/jacs.5c12199 (PMC12550841; doi:10.1021/jacs.5c12199)
Supplement: Supplementary file 1 [file ja5c12199_si_001.pdf]

Supporting Information

Mechanistic Dissymmetry between Crystal Growth and Dissolution drives Ratcheted Chiral Amplification

Sjoerd W. van Dongen[a†], Jin Maeda[b,c†], Bernard Kaptein[d], Pascal Cardinael[b], Adrian Flood[c*], Gerard Coquerel[b*] and Willem L. Noorduin[a,e*]

[a] AMOLF, Science Park 104, 1098 XG, Amsterdam, The Netherlands.

[b] Univ Rouen Normandie, Normandie Univ, SMS UR 3233, F-76000 Rouen, France.

[c] School of Energy Science and Engineering, Vidyasirimedhi Institute of Science and Technology, Rayong 21210, Thailand.

[d] InnoSyn, Urmonderbaan 22, 6167 RD, Geleen, The Netherlands.

[e] Van ‘t Hoff Institute for Molecular Sciences, University of Amsterdam, Science Park 904, 1098 XH, Amsterdam, The Netherlands.

* Email: adrian.flood@vistec.ac.th; gerard.coquerel@univ-rouen.fr; noorduin@amolf.nl
† Authors contributed equally

Table of Contents

Experimental Details p. 2

General Materials and Methods p. 2

Materials p. 2

HPLC Analysis p. 2

*Solubility of **1*** p. 2

*Solubility of **2*** p. 2

Deconvoluted temperature cycles p. 3

Experimental set-up and sampling p. 3

Deconvoluted temperature cycles for low, medium, high initial ee p. 4

Heating rate and isothermal hold p. 4

Relative amount of seed crystals p. 4

Dissymmetry between dissolution and growth p. 5

Experimental set-up and sampling p. 5

Isolated dissolution p. 5

Isolated growth p. 5

Mass balance and ee_{Δ} p. 6

Effect of racemization rate p. 6

One-time switching off racemization in a temperature cycle p. 7

Thermodynamic Analysis using Phase Diagrams p. 7

Model for Asymmetric Growth and Dissolution p. 8

References p. 12

Experimental Details

General Materials and Methods

Materials

Paclobutrazol precursor **1** (1-(4-Chlorophenyl)-4,4-dimethyl-2-(1H-1,2,4-triazol-1-yl)pentan-3-one, called Cl-TAK) was synthesized as per previously reported methods [1, 2, 3]. Tert-leucine precursor **2** (3,3-dimethyl-2-((naphthalen-2-ylmethylene)amino)butanenitrile) was obtained over the course of previous research [4]. Solvents, reagents, and analytical-grade chemicals were purchased from commercial suppliers and used as received unless otherwise specified. Sodium hydroxide pellets (85.0–100.5%, VWR Chemicals), 1,8-diazabicyclo[5.4.0]undec-7-ene (98+%, Acros Organics), anisole (99%, Thermo Scientific Chemicals), methanol (99%, Thermo Scientific Chemicals), and demineralized water were used in this study.

HPLC Analysis

Enantiomeric excess of **1** was determined using a Thermo Scientific UltiMate 3000 chiral HPLC system equipped with a Chiralcel OD-H column (4.6 mm × 250 mm, 5 μm) and UV detection at 220 nm. The mobile phase consisted of n-heptane:2-propanol (80:20, v/v) at a flow rate of 1.0 mL/min, with retention times of 7.4 min for (R)-**1** and 9.2 min for (S)-**1**. Chiral HPLC analyses of **2** were performed using an Agilent Technologies Infinity 1260 HPLC system equipped with a CHIRALPAK IA column (250 x 4.6 mm, 5μm) and UV detection at 220 nm. The mobile phase consisted of n-heptane:2-propanol (95:5, v/v) at a flow rate of 0.7 mL/min, with retention times of 5.2 min for anisole (internal standard), 7.9 min for (S)-**2**, and 9.1 min for (R)-**2**. All solvents used (n-heptane, 2-propanol) were HPLC grade (≥ 99%) and obtained from VWR chemicals.

*Solubility of **1***

The solubility of racemic **1** in MeOH:Water (60:40, v/v) was determined using a Technobis Crystal16 instrument (Alkmaar, The Netherlands) and is displayed in Figure S-1 (figure on the next page).

Masses ranging from 5 to 62 mg of racemic **2** were placed in approximately 900 mg of 60 wt% methanol-water. Controlled temperature ramps from −5 °C to 60 °C were performed and repeated three times, while the turbidity was monitored to determine the clear points (solubility curve). The Van 't Hoff equation was fitted to the data between 10 °C and 60 °C.

Specifically, all samples were fully dissolved at 60 °C, where the temperature program began by cooling to -5 °C at a rate of 0.5 °C/min (2 hours and 10 minutes), followed by an isothermal hold for 5 hours. The temperature was then increased to 60 °C at 0.3 °C/min (3 hours and 36 minutes) with a final isothermal hold of 30 minutes. The solubility temperature for each sample was determined as the temperature at which transmissivity reached 100% (clear solution) during the heating cycle.

This experiment was conducted both with and without NaOH (0.1 wt% of solvent), and no significant differences in solubility temperatures were observed between the two conditions. Additionally, no significant variations were detected across the three repeated temperature cycles, demonstrating the robustness of the measurements.

*Solubility of **2***

The solubility curve and metastable zone of racemic **2** in MeOH were similarly determined using the Technobis Crystal16 instrument and are displayed in Figure S-2 (temperature range: 15 – 40 ºC). The metastable zone was determined by also monitoring the cloud points (nucleation indicates end of metastable zone). The effect of racemization catalyst DBU was also assessed, showing constant elevated solubility due to the formed racemization intermediates (Figure S-2; figure on the next page).

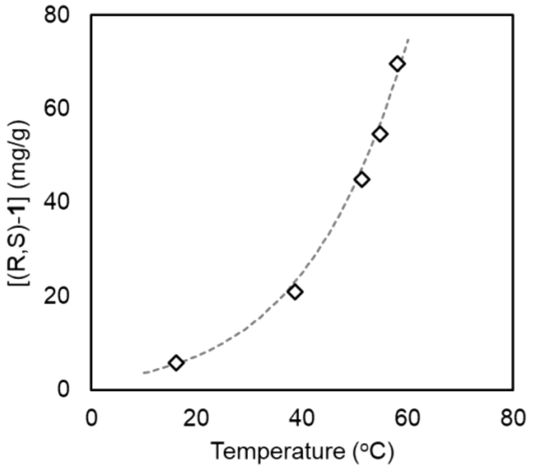

Figure S-1. Temperature dependent solubility (diamonds) of racemic **1** in MeOH:Water (60:40, v/v). Van 't Hoff fit shown with grey dotted line.

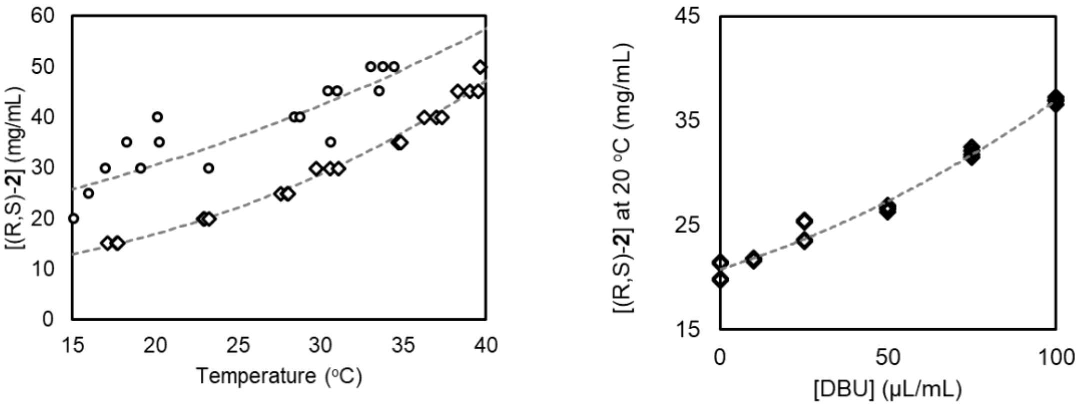

Figure S-2. Temperature dependent solubility (diamonds) and metastable zone (circles) for racemic **2** in MeOH (left) and effect of [DBU] on the room temperature solubility (right). Van 't Hoff fit shown with grey dotted line.

Deconvoluted temperature cycles

Experimental set-up and sampling

All experiments were conducted in 50 mL jacketed round-bottom flasks equipped with a magnetic stir bar and connected to a LAUDA ECO RE 630 S thermostat for precise temperature control. The stirring rate was set at 500 rpm. Methanol/water (60 wt% methanol) was chosen as the solvent system due to its optimal solubility profile of **1**. Racemization was achieved by adding NaOH (0.1 wt% of solvent).

Samples were taken at specific time points during the TCID (temperature cycling-induced deracemization, i.e. cycles of heating and cooling to induce dissolution and growth) cycles: (1) before initiating heating to determine the initial solid phase enantiomeric excess, (2) after the isothermal hold following dissolution, and (3) after the isothermal hold following growth. Approximately 0.3 mL of suspension was withdrawn using a plastic pipette. To prevent racemization during analysis, the solids were rapidly filtered, washed with aqueous HCl (6 M), and subsequently dissolved in methanol for analysis.

The seeds for temperature cycling are prepared by dissolving a mixture of racemic and enantiopure **1** (total 2.7 g) in methanol (18 g) preheated to 40 °C. Deionized water (11.8 g) was then added dropwise

to induce antisolvent crystallization. Racemization was initiated by introducing NaOH to the system (0.1 wt% of solvent).

Deconvoluted temperature cycles for low, medium, high initial ee

The deconvoluted temperature cycle is initiated by starting the temperature program. The temperature cycle consisted of sampling, heating from 40 °C to 55 °C at 1.5 °C/min, holding isothermally for 10 minutes, sampling, cooling back to 40 °C at 0.5 °C/min, and holding isothermally for 10 minutes, and sampling; resulting in a total cycle time of 60 minutes. During a cycle, 50% of the initial solids dissolve and regrow.

The effects of the initial solid phase ee were investigated by varying the initial enantiomeric excess of the seed-slurry of **1** in each experiment. Three levels of initial ee were used: low (weighed 25%, resulting in an initial 20–30% after antisolvent crystallization), medium (50, resulting in 50–55% after antisolvent crystallization), and high (90%, resulting in 80–90% after antisolvent crystallization).

Heating rate and isothermal hold

To exclude any effect from the heating rate, we repeated the cycle step for slurries starting with high initial ee but with fast (3 °C/min) and slow (0.25 °C/min) heating rate during the dissolution step (Table S-1). The effect of different heating rate was negligible, showing that choice of heating rate did not significantly affect the asymmetric dissolution or growth.

Table S-1. Effect of heating rate on cycling of **1**, starting with high initial *ee* in (R)-**1**.

	5 min. (3 °C/min)	60 min. (0.25 °C/min)
Before Heating	98.72%	98.92%
After Heating	91.79%	89.62%
After Cooling	98.92%	99.00%

In addition, we investigated the effect of the length of the isothermal hold after the dissolution segment. In this experiment, we tested a short (5 min) and long (55 min) isothermal hold at 55 °C and measured the solid phase *ee* in both experiments (Table S-2). No significant difference is observed between the experiments, and a sharp decrease in *ee* due to antagonistic effects of dissolution is observed immediately.

Table S-2. Effect of isothermal hold on dissolution of **1**, starting with high initial *ee* in (R)-**1**.

	solid *ee* (%)
Before heating (40 °C)	99.1
5 minutes after reaching 55°C	91.6
55 minutes after reaching 55°C	92.1
After cooling (40°C)	99.8

Relative amount of seed crystals

We also varied the total mass of **1** in each experiment while keeping the total solvent mass constant. This approach ensured control over the percentage of the suspension that underwent dissolution and crystallization at the start of each experiment. Three groups were defined based on the percentage of the suspension exchanged per temperature cycle: low (30%), medium (50%), and high (80%). The total mass of **1** was adjusted to achieve these percentages, with 4.00 grams used for the low group, 2.70 grams for the medium group, and 1.95 grams for the high group.

We expect that increasing the relative amount of material that is dissolved and grown makes the asymmetric effects more pronounced [5]. We repeated the deconvoluted temperature cycle experiments wherein we dissolve and regrow 80% of the solids rather than the 50% in the previous experiments. As shown in Table S-3, the asymmetric effects become more pronounced if the relative cyclic mass transfer is increased. Dissolution has a stronger antagonistic effect and growth has a stronger agonistic effect on chiral amplification when the relative amount of dissolved and grown material is increased.

Table S-3. Effect of relative amount of seed crystals on asymmetric dissolution and growth of **1**, controlled through varying the relative amount of solids that dissolve and grow (cyclic mass transfer) during the deconvoluted temperature cycle experiment.

	low initial ee		medium initial ee		high initial ee	
rel. cyclic mass transfer	50%	80%	50%	80%	50%	80%
ee before dissolution (%)	27.9	31.8	56.6	51.3	91.9	85.8
ee after dissolution (%)	55.4	39.7	80.8	43.0	87.8	66.7
ee after growth (%)	47.8	66.8	84.3	97.4	97.5	96.2

Dissymmetry between dissolution and growth

Experimental set-up and sampling

Experiments were conducted in 7 mL vials sourced from Merck (27150-U Supelco). An aluminum block with milled holes for vials was fabricated in-house and placed on top of a shaker (VWR Standard vortex mixer). Temperature was controlled through a thermostat (Huber Heat Regulator RS2232, range: 5 – 60 °C). To ensure homogenization and minimize attrition and secondary nucleation, we used PTFE beads (3 mm PTFE balls, BOLA). To the solvent (MeOH), we added an internal standard (0.2 wt% anisole) to allow the determination of liquid phase concentration in samples to ascertain a full mass balance.

For seed crystal preparation, a 20 mL vial was charged with 5 g of glass beads (2 mm glasschrot, through VWR), 750 mg of **2** (~50% ee in (R)-**2**, by mixing enantiopure (R)-**2** and racemic **2**), and 5 mL 2-propanol. The vial was sonicated for 5 hours at low temperature (10 °C). After sonication, the vial was allowed to equilibrate in the fridge, before filtration over a P4 glass filter afforded a fine and homogeneous white solid. The *ee* of the obtained seed crystals was determined by HPLC analysis to be 50.89% in (R)-**2**.

For sampling, we now discriminated between liquid and solid phase sampling. For liquid phase samples: 50 – 100 µL of liquid was obtained by filtering 125 µL of slurry over a syringe filter (0.2 µm PTFE, VWR International) and mixing it with 1 mL of 2-propanol by vortex. The solution was then submitted for HPLC analysis. For solid phase samples, aliquots of 0.5 – 1.0 mg of solid material were collected from the solids obtained by filtering 250 µL slurry over a P4 glass filter under vacuum. The solids were dissolved in 1.5 mL of 2-propanol by vortex and subsequent ultrasonication (10 minutes) and then submitted for HPLC analysis.

Isolated dissolution

For isolated dissolution, a 7 mL vial was charged with 27 mg of seed crystals and 0.7 g of PTFE spheres. Subsequently, an undersaturated solution of racemic **2** was prepared by dissolving 12 mg (R,S)-**2** in 1 mL of MeOH (with 0.2 wt% anisole) at 20 °C in 1 mL and adding 10 µL/mL racemization catalyst (DBU). The liquid phase of the solution was analyzed. The vial with the crystal was placed in the shaker and shaking was initiated. The 1 mL solution was added to the crystals under shaking to induce instantaneous dissolution. After 90 minutes, the solid and liquid phases of the resulting slurry were analyzed.

Isolated growth

For isolated growth, a 7 mL vial was charged with 20 mg of seed crystals and 0.7 g of PTFE spheres. Subsequently, a saturated solution of racemic **2** was prepared at 30 °C in the presence of 10 µL/mL racemization catalyst (DBU). The liquid phase of the solution was analyzed. The vial with the crystal was placed in the shaker, kept at 30 °C, and shaking was initiated. The 1 mL solution was added to the

crystals under shaking. The shaker was cooled back to 20 °C over the course of 90 minutes to induce growth. After 30 minutes, the solid and liquid phases of the resulting slurry were analyzed.

Mass balance and ee_Δ

As shown in [5], the enantiomeric excess of grown (or dissolved) material (ee_Δ) can be calculated if one knows the *ee* of the initial solid (seed crystals) denoted ee_0, the *ee* of the material after growth (or dissolution) denoted ee_P, the mass of the seed crystals (denoted m_0), and the mass change during growth or dissolution (denoted m_Δ). The equation to calculate ee_Δ is as follows:

$$ee_\Delta = ee_P + \frac{m_0}{m_\Delta}(ee_P - ee_0) \qquad \text{(eq. 1)}$$

In our experiments, m_0 (weighed seed amount) and ee_0 (seed composition) are known at the start of the experiment. After growth or dissolution, ee_P is determined by analyzing the resulting solids through chiral HPLC. Finally, m_Δ is obtained by comparing liquid phase concentration of the initial solution (c_{initial}) that is added to the seed crystals and the concentration of the liquid phase of the slurry after growth or dissolution (c_{final}) as follows:

$$m_\Delta = c_{\text{initial}} - c_{\text{final}} \qquad \text{(eq. 2)}$$

We determine these concentrations through quantitative HPLC. The solvent contains anisole as internal standard, so that the concentration of (R)-2 and (S)-2 in solution can be quantified (Figure S-3).

This full mass balance hence allows calculating the exact ee_Δ after growth and dissolution to yield insight in what happens to the crystals during the process, rather than only viewing the initial and final *ee*.

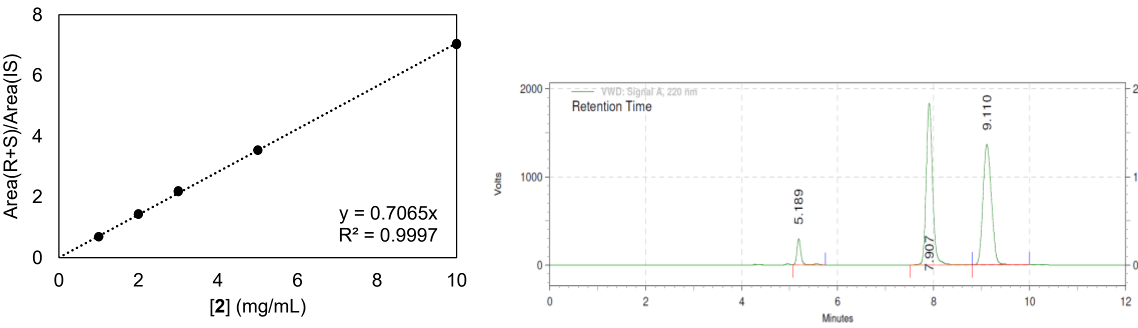

Figure S-3. Calibration curve (left) for racemic **2** in MeOH with 0.2wt% anisole as internal standard, obtained from chiral HPLC (right). A quantifier for concentration is achieved by taking the peak areas of the enantiomers (7.9 min for (S)-**2**, and 9.1 min for (R)-**2**) relative to the peak area of the internal standard (5.2 min for anisole).

Table S-4. Full mass balance results with determined values of ee_0, ee_Δ, and final *ee*.

		dissolution			growth		
		solid mass R (mg/mL)	solid mass S (mg/mL)	*ee*	solid mass R (mg/mL)	solid mass S (mg/mL)	*ee*
	initial (0)	20.37	6.63	50.9%	15.09	4.91	50.9%
[Fig. 3b]	final	14.80	2.38	72.3%	22.80	10.43	37.2%
[Fig. 3d]	change (Δ)	-5.57	-4.25	13.5%	7.71	5.52	16.6%

Effect of racemization rate

To assert the effect of racemization rate, the isolated dissolution and growth experiments were repeated at various concentrations of racemization catalyst (DBU): 0, 10, 25, 50, 75 µL/mL for dissolution and 5, 15, 25, 50 µL/mL for growth. The initial amount of seed crystals was as described previously, but the concentration of the undersaturated solution for dissolution was adapted to the changed solubility due to changed DBU concentration (11, 13.5, 16, 18.5 mg/mL for 10, 25, 50, 75 µL/mL DBU respectively).

<u>One-time switching off racemization in a temperature cycle</u>

Two identical TCID experiments of **1** were set up with an initial solid phase ee of 20% in (R)-**1** and temperature cycling was induced for three full cycles to reach a solid phase ee of 40 – 45% in (R)-**1**. After four more temperature cycles (parameters as before), one experiment proceeded without modification, while in the other, racemization was switched off. To achieve this, the temperature program was paused at 40 °C, and 6 mL of 6 M aq. HCl solution was added in excess to fully neutralize NaOH and ensure no racemization occurred. Neutralization was confirmed by a color change from dark blue to red on pH paper. The suspension was then stirred isothermally for 10 minutes before resuming the temperature program (heating segment for final non-racemizing dissolution step).Before the cooling segment to induce the final growth step, racemization catalyst was re-added (0.2 wt% NaOH).

Thermodynamic Analysis using Phase Diagrams

To evaluate the applicability of conventional thermodynamic models to TCID, experimental results were compared to predictions based on ternary phase diagrams. These diagrams describe the phase behavior of enantiomers under equilibrium conditions, assuming ideal mixing and no kinetic effects.

In a non-racemizing solution, both enantiomers dissolve according to their solubilities at a given temperature. If a scalemic solid mixture is heated, dissolution occurs until the system reaches equilibrium with the saturated solution phase. In some cases, if the minor enantiomer dissolves completely before equilibrium is reached, the remaining solid phase consists entirely of the major enantiomer, and the solution exhibits an excess of the major enantiomer after dissolution. Conventional ternary phase diagrams predict that heating a scalemic mixture should generally result in an increase in enantiomeric excess of the solid phase as dissolution progresses.

However, experimental results show significant and repeatable deviations from these predictions in the presence of instantaneous racemization. In a racemizing solution, the solution cannot sustain an enantiomeric excess, as racemization continuously interconverts enantiomers in solution (Figure S-4). This fundamentally alters the accessible phase space in the ternary system, preventing the expected increase in enantiomeric excess upon heating (Figure S-5). Instead, in several observed cases, a decrease in enantiomeric excess occurs experimentally, demonstrating that ternary phase diagrams alone cannot always accurately describe systems undergoing active racemization (Figure S-6).

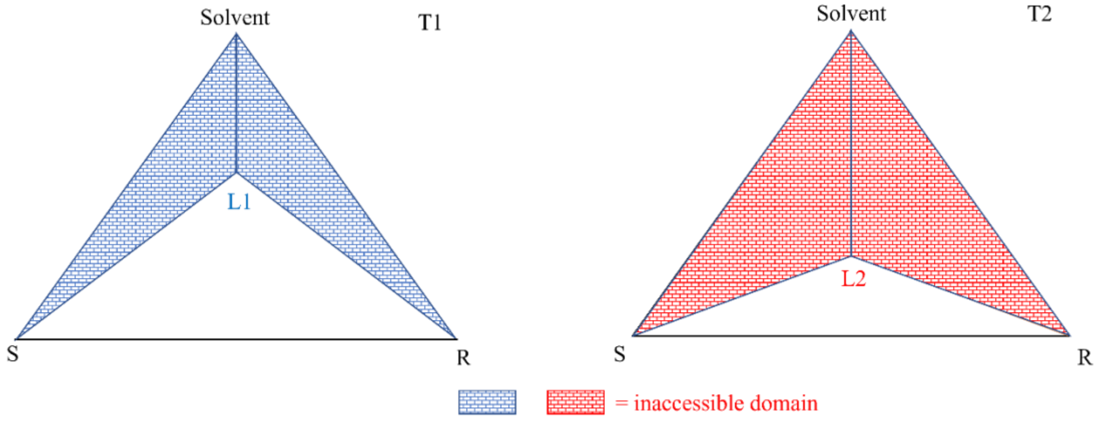

Figure S-4. Theoretical Ternary Isotherms of a conglomerate forming system at T1 and T2 with T2>T1 and instant racemization in the solution. Due to the instant racemization in solution, phases can only exist along the segment represented by the pure solvent and point L1/L2 and in the white triangle L1/L2—<S>—<R>.

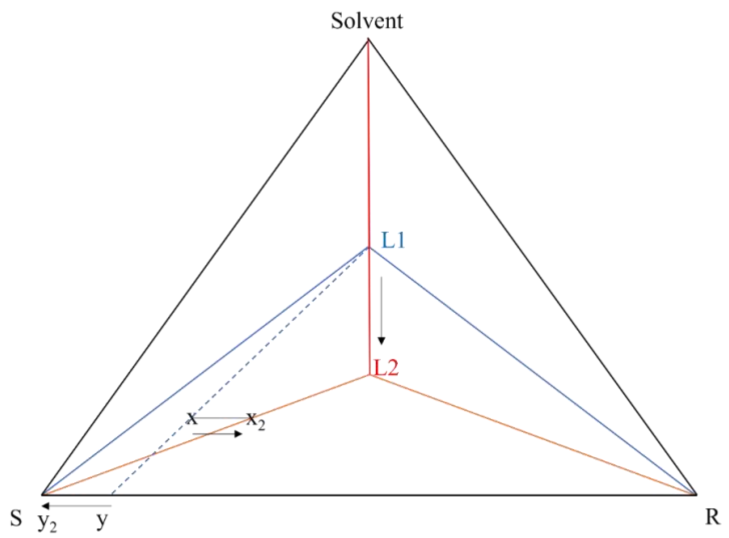

Figure S-5. Theoretical evolution on heating described by the superimposition of two ternary isotherms at T1 and T2 where T2 > T1 and instant racemization in the liquid state. The overall composition of x at T1 gives solid composition of y, and shifts to x2 and y2 (pure enantiomer) at T2.

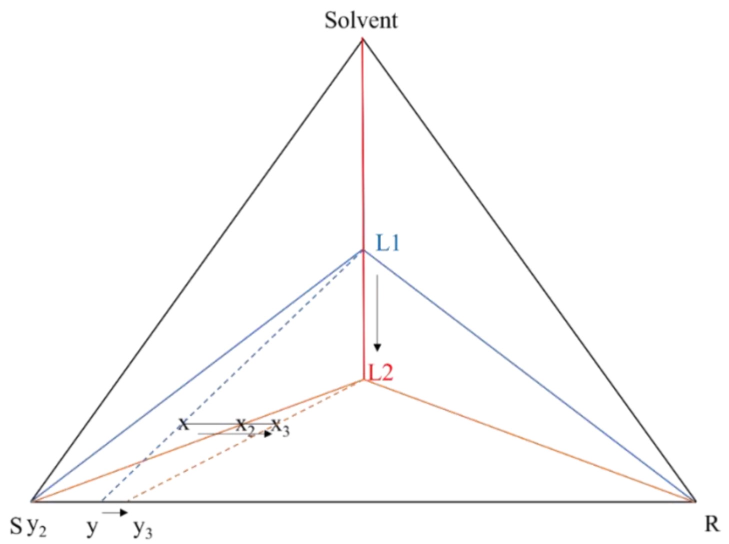

Figure S-6. Experimentally observed evolution on heating described by the superimposition of two ternary isotherms at T1 and T2 where T2>T1 and instant racemization in the liquid state. The overall composition of x at T1 shifts to x3 instead of x2, resulting in a decreased solid composition of y3.

Note: The inaccessible domains in Figures S-5 and S-6 are not shaded to improve legibility.

Model for asymmetric growth and dissolution

We adapt the empirical model for chiral amplification through asymmetric crystal growth under racemizing conditions presented in [5], that is based on a so-called amplification factor and makes no further assumptions on the underlying source of chiral amplification.

In short, the model empirically describes the extent of asymmetry during a growth or dissolution step using the amplification factor α defined by

$$\alpha = \frac{ee_\Delta}{ee_0} \qquad \text{(eq. 3)}$$

One then rewrites eq. 1 and 3 to yield a description of the evolution of solid phase *ee* after a dissolution or growth step as:

$$ee_P = ee_0 \cdot \left(\frac{\frac{m_0}{m_\Delta} + \alpha}{\frac{m_0}{m_\Delta} + 1} \right) \qquad \text{(eq. 4)}$$

This equation was numerically implemented by calculating the evolution of *ee* as a function of cycles consisting of an initial dissolution segment (with a specific value of α for dissolution) and a subsequent growth segment (with a specific value of α for growth). The final *ee* of the growth segment was taken as the ee_0 for the next cycle. Note that for the growth segment, $m_0 \to m_0 - m_\Delta$.

As values for the two amplification factors (which were assumed to be independent of ee_0, since this was the case in [5]), we used the predicted plateaus of **2** to estimate $\alpha_{\text{disolution}} = 0.6$ and $\alpha_{\text{growth}} = 0.8$. These were the closest values we could relate to our experiments here. Of course, in reality, a sigmoidal shape would be expected for kinetics since size distributions change and α will disperse with evolving crystal populations and *ee*. We then ran numerical calculations using $ee_0 = 0.1$, $m_0 = 27$ and $m_\Delta = 7$, although these only affect scaling.

To assess the effect of switching on and off racemization during dissolution or growth segments, we set $\alpha_{\text{disolution}} = 0$ or $\alpha_{\text{growth}} = 0$ respecitvely.

The script used to perform the numerical calculations, using software package "R" [6], is provided below as Script S-1.

Script S-1. Numerical implementation of the adapted model in R [6].

```
# Functions
calculateEE <- function(R, S){
  return((R - S)/(R + S))
}
calculateR <- function(ee, m){
  return(0.5*(ee + 1)*m)
}
calculateS <- function(ee, m){
  return(m - calculateR(ee, m))
}

# Parameters
ee0 = 0.1
m0 = 27
mD = 7
alfaG = 0.8
alfaD = 0.6
steps = 30

# Simulation
df <- data.frame(
  iteration = numeric(steps*2+ 1),  # Iteration
  regR = numeric(steps*2+ 1),    # R for regular TC
  regS = numeric(steps*2+ 1),     # S for regular TC
  regee = numeric(steps*2+ 1),     # ee for regular TC
  grOnlyR = numeric(steps*2+ 1),      # R for TC with racemization during growth only
  grOnlyS = numeric(steps*2+ 1),   # S for TC with racemization during growth only
  grOnlyee = numeric(steps*2+ 1),   # ee for TC with racemization during growth only
  disOnlyR = numeric(steps*2+ 1),       # R for TC with racemization during dissolution only
```

```
  disOnlyS = numeric(steps*2+ 1),     # S for TC with racemization during dissolution only
  disOnlyee = numeric(steps*2+ 1)     # ee for TC with racemization during dissolution only
)

# Initial conditions
df$iteration = seq(0, steps, 0.5)
R0 = calculateR(ee0, m0)
S0 = calculateS(ee0, m0)
df[1,c(4,7,10)] <- ee0
df[1,c(2,5,8)] <- R0
df[1,c(3,6,9)] <- S0

for (i in 2:(steps*2 + 1)) {
  if(i %% 2 == 0){
    # B. Growth Step
    # Regular TC
    eeD = alfaG*df$regee[i-1]
    if(eeD > 1){
      eeD = 1
    }
    R0 = calculateR(df$regee[i-1], m0-mD)
    S0 = calculateS(df$regee[i-1], m0-mD)
    deltaR = calculateR(eeD, mD)
    deltaS = calculateS(eeD, mD)
    Rnew = R0 + deltaR
    Snew = S0 + deltaS
    eenew = calculateEE(Rnew, Snew)
    df$regR[i] = Rnew
    df$regS[i] = Snew
    df$regee[i] = eenew

    # Rac. Growth only
    eeD = alfaG*df$grOnlyee[i-1]
    if(eeD > 1){
      eeD = 1
    }
    R0 = calculateR(df$grOnlyee[i-1], m0-mD)
    S0 = calculateS(df$grOnlyee[i-1], m0-mD)
    deltaR = calculateR(eeD, mD)
    deltaS = calculateS(eeD, mD)
    Rnew = R0 + deltaR
    Snew = S0 + deltaS
    eenew = calculateEE(Rnew, Snew)
    df$grOnlyR[i] = Rnew
    df$grOnlyS[i] = Snew
    df$grOnlyee[i] = eenew

    # Rac. Dissolution only
    eeD = 0
    R0 = calculateR(df$disOnlyee[i-1], m0-mD)
    S0 = calculateS(df$disOnlyee[i-1], m0-mD)
    deltaR = calculateR(eeD, mD)
    deltaS = calculateS(eeD, mD)
    Rnew = R0 + deltaR
    Snew = S0 + deltaS
    eenew = calculateEE(Rnew, Snew)
```

```
    df$disOnlyR[i] = Rnew
    df$disOnlyS[i] = Snew
    df$disOnlyee[i] = eenew

  }else{
    # A. Dissolution Step
    # Regular TC
    eeD = alfaD*df$regee[i-1]
    if(eeD > 1){
      eeD = 1
    }
    R0 = calculateR(df$regee[i-1], m0)
    S0 = calculateS(df$regee[i-1], m0)
    deltaR = calculateR(eeD, mD)
    deltaS = calculateS(eeD, mD)
    if(deltaR > R0){
      deltaS = deltaS + deltaR - R0
      deltaR = R0
    }
    if(deltaS > S0){
      deltaR = deltaR + deltaS - S0
      deltaS = S0
    }
    Rnew = R0 - deltaR
    Snew = S0 - deltaS
    eenew = calculateEE(Rnew, Snew)
    df$regR[i] = Rnew
    df$regS[i] = Snew
    df$regee[i] = eenew

    # Rac. Growth only
    eeD = 0
    R0 = calculateR(df$grOnlyee[i-1], m0)
    S0 = calculateS(df$grOnlyee[i-1], m0)
    deltaR = calculateR(eeD, mD)
    deltaS = calculateS(eeD, mD)
    if(deltaR > R0){
      deltaS = deltaS + deltaR - R0
      deltaR = R0
    }
    if(deltaS > S0){
      deltaR = deltaR + deltaS - S0
      deltaS = S0
    }
    Rnew = R0 - deltaR
    Snew = S0 - deltaS
    eenew = calculateEE(Rnew, Snew)
    df$grOnlyR[i] = Rnew
    df$grOnlyS[i] = Snew
    df$grOnlyee[i] = eenew

    # Rac. Dissolution only
    eeD = alfaD*df$disOnlyee[i-1]
    if(eeD > 1){
      eeD = 1
    }
```

```
    R0 = calculateR(df$disOnlyee[i-1], m0)
    S0 = calculateS(df$disOnlyee[i-1], m0)
    deltaR = calculateR(eeD, mD)
    deltaS = calculateS(eeD, mD)
    if(deltaR > R0){
      deltaS = deltaS + deltaR - R0
      deltaR = R0
    }
    if(deltaS > S0){
      deltaR = deltaR + deltaS - S0
      deltaS = S0
    }
    Rnew = R0 - deltaR
    Snew = S0 - deltaS
    eenew = calculateEE(Rnew, Snew)
    df$disOnlyR[i] = Rnew
    df$disOnlyS[i] = Snew
    df$disOnlyee[i] = eenew

  }
}

# Create Export
dfExport <- df[df$iteration %% 1 == 0, !(names(df) %in% c("regR", "regS", "grOnlyR", "grOnlyS",
"disOnlyR", "disOnlyS"))]
write.csv(dfExport, "exportedDataSimulation.csv", row.names = TRUE)
```

References

[1] Lopes, C.; Cartigny, Y.; Brandel, C.; Shemchuk, O.; Leyssens, T. A Greener Pathway to Enantiopurity: Mechanochemical Deracemization through Abrasive Grinding. *Chem. Eur. J.* **2023**, 29 (40), e202300585. DOI: 10.1002/chem.202300585.

[2] Bovonsombat, P.; Teecomegaet, P.; Kulvaranon, P.; Pandey, A.; Chobtumskul, K.; Tungsirisurp, S.; Sophanpanichkul, P.; Losuwanakul, S.; Soimaneewan, D.; Kanjanwongpaisan, P.; Siricharoensang, P.; Choosakoonkriang, S. Regioselective Monobromination of Aromatics via a Halogen Bond Acceptor-Donor Interaction of Catalytic Thioamide and N-Bromosuccinimide. *Tetrahedron* **2017**, 73 (46), 6631–6639. DOI: 10.1016/j.tet.2017.10.005.

[3] Qizhou, J. (Green Chemical Co., Ltd). Preparation Method of Chlorazolam. Chinese Patent CN 111777565 A, **2020**.

[4] Baglai, I.; Leeman, M.; Wurst, K.; Kaptein, B.; Kellogg, R.M.; Noorduin. W.L. The Strecker reaction coupled to Viedma ripening: a simple route to highly hindered enantiomerically pure amino acids. *Chem. Commun.* **2018**, 54, 10832–10834. DOI: 10.1039/C8CC06658B.

[5] Van Dongen, S. W., et al. Chiral Amplification through the Interplay of Racemizing Conditions and Asymmetric Crystal Growth. *J. Am. Chem. Soc.* **2023** 145 (1), 436–442.

[6] R Core Team (**2021**). R: A language and environment for statistical computing. R Foundation for Statistical Computing, Vienna, Austria. https://www.R-project.org/.
